# Update of Endocrine Dysfunction following Pediatric Traumatic Brain Injury

**DOI:** 10.3390/jcm4081536

**Published:** 2015-07-31

**Authors:** Kent Reifschneider, Bethany A. Auble, Susan R. Rose

**Affiliations:** 1 Children’s Hospital of The Kings Daughters, Eastern Virginia Medical School, Norfolk, Virginia, VA 23507, USA; E-Mail: Kent.Reifschneider@chkd.org; 2 Children’s Hospital of Wisconsin, Medical College of Wisconsin, Milwaukee, Wisconsin, WI 53226, USA; E-Mail: bauble@mcw.edu; 3 Cincinnati Children’s Hospital Medical Center and College of Medicine, University of Cincinnati, MD, 3333 Burnet Avenue, MLC 7012, Cincinnati, OH 45229, USA

**Keywords:** traumatic brain injury, hypopituitarism, precocious puberty, hypogonadotropic hypogonadism, central hypothyroidism, growth hormone deficiency, adrenal insufficiency, hyperprolactinemia, adult, pediatric

## Abstract

Traumatic brain injuries (TBI) are common occurrences in childhood, often resulting in long term, life altering consequences. Research into endocrine sequelae following injury has gained attention; however, there are few studies in children. This paper reviews the pathophysiology and current literature documenting risk for endocrine dysfunction in children suffering from TBI. Primary injury following TBI often results in disruption of the hypothalamic-pituitary-adrenal axis and antidiuretic hormone production and release, with implications for both acute management and survival. Secondary injuries, occurring hours to weeks after TBI, result in both temporary and permanent alterations in pituitary function. At five years after moderate to severe TBI, nearly 30% of children suffer from hypopituitarism. Growth hormone deficiency and disturbances in puberty are the most common; however, any part of the hypothalamic-pituitary axis can be affected. In addition, endocrine abnormalities can improve or worsen with time, having a significant impact on children’s quality of life both acutely and chronically. Since primary and secondary injuries from TBI commonly result in transient or permanent hypopituitarism, we conclude that survivors should undergo serial screening for possible endocrine disturbances. High indices of suspicion for life threatening endocrine deficiencies should be maintained during acute care. Additionally, survivors of TBI should undergo endocrine surveillance by 6–12 months after injury, and then yearly, to ensure early detection of deficiencies in hormonal production that can substantially influence growth, puberty and quality of life.

## 1. Introduction

In the United States, pediatric traumatic brain injury (TBI) places a significant burden on our health care system. TBI is the leading cause of death and disability in industrialized countries. In the USA, more than 17 million TBIs occur annually, with at least 5.3 million Americans living with disabilities due to sequelae [1]. Permanent disabilities (neurocognitive and/or endocrine) develop in 10% of adults who suffered a mild TBI, and in 66% and 99% after moderate and severe TBI respectively [2]. Among adults ages 25–64 years, the rates for TBI-related emergency hospitalizations have reached 72 per 100,000 [3]. Although hospitalizations for children decreased slightly in the last decade, in 2009–2010 54 per 100,000 persons under the age of 24 years suffered a significant TBI requiring hospitalization [3]. Most commonly affected are young adults (ages 15–24) primarily due to motor vehicle accidents, followed by ages 0–4 suffering falls, with males affected more than females [3]. Approximately 35% of affected children suffer poor cognitive and functional outcomes two years after injury [3]. In prospective evaluation, at least 40% of children still had impaired quality of life at one year after moderate to severe head injury [4]. Recently, the number of publications addressing endocrine function after TBI in adults has increased; leading to awareness that pituitary dysfunction is a common sequalae [5,6,7,8,9,10,11]. In contrast, pediatric literature analyzing endocrine function after TBI in children remains sparse. Additionally, the lack of consensus guidelines for endocrine stimulation testing, preferred hormone assays, diagnostic criteria, and optimal duration from time of injury until evaluation, further complicates the ability to understand the frequency of endocrine dysfunction following TBI.

The purpose of this review is to summarize and analyze the pediatric literature to date regarding endocrine function after TBI, and to propose guidelines for clinical monitoring.

## 2. Mechanism of Injury during Traumatic Brain Injuries

**Figure 1:**
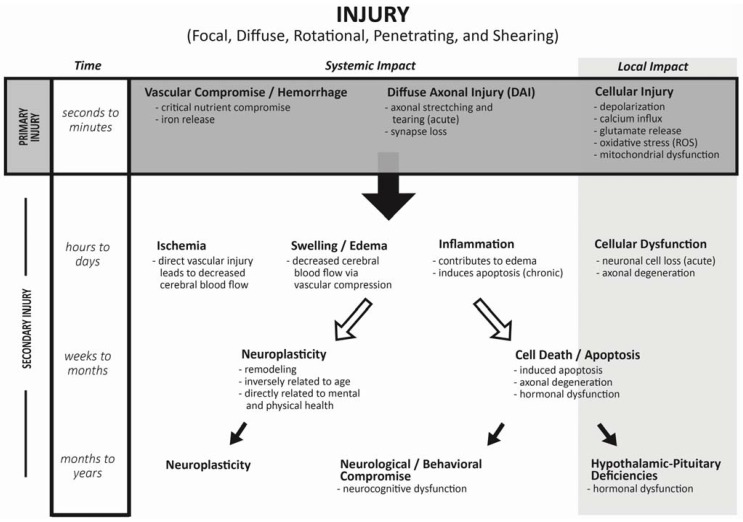
Hypothesized model for progression from Primary to Secondary Injury after trauma to the central nervous system.

Traumatic brain injury is described as a non-congenital insult to the brain from an external mechanical force causing temporary or permanent neurological dysfunction. TBI is classified as focal due to direct physical impact to the brain (penetrating or contusion), or diffuse following rapidly changing forces. The initial injury or mechanism can include penetrating, tearing, shearing, and/or hemorrhage. Initial physiologic response to TBI, or “primary injury”, involves decrease in cerebral flow leading to decreased protein synthesis and acidosis. Increases in free radical and cation production alter the electro-potential gradient, causing fluid shifts and resulting in swelling and apoptosis (Figure 1).

**Figure 2:**
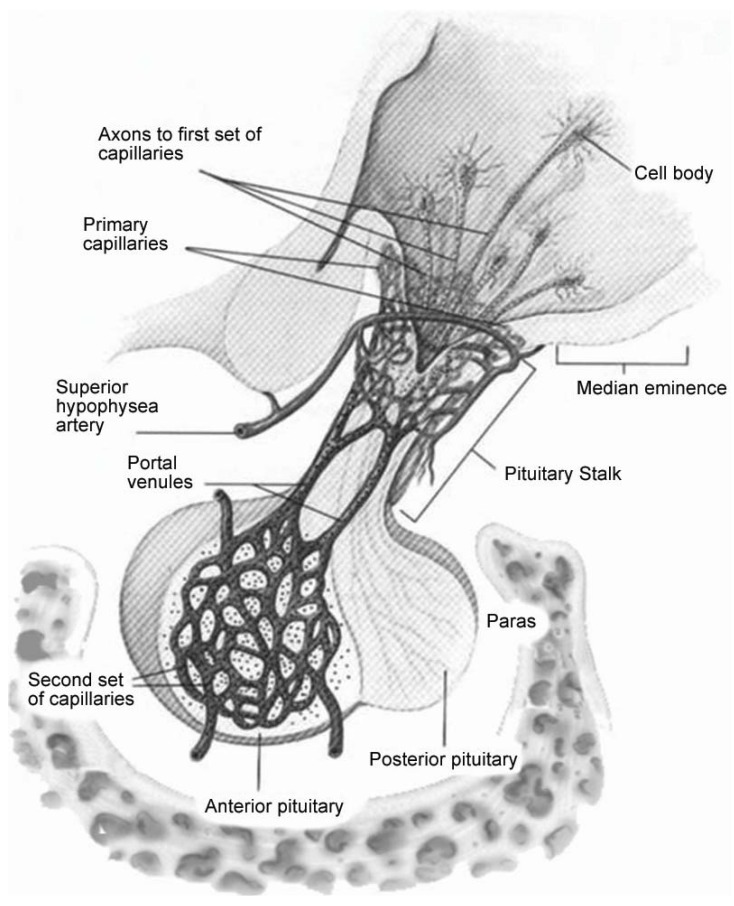
The pituitary gland is suspended via the infundibulum. Long hypophyseal blood vessels surround the pituitary stalk, supplying the anterior pituitary with small perforating vessels. The surrounding vessels and axons are susceptible to shearing from external forces and swelling [13].

The subsequent reaction to the primary injury (“secondary injury”) is thought to be far more damaging to the surrounding tissue. Secondary injury typically develops within hours or days to weeks following the primary injury [12]. Swelling further compromises vascular support causing distal tissue to be at greater risk of temporary or permanent cell death. Globally, central nervous system (CNS) swelling leads to risk of cerebellar tonsillar herniation which can compromise the respiratory drive center within the midbrain. Similarly, swelling near the hypothalamus compromises the infundibulum that is vital for pituitary vascular supply, communication and function. The long hypophyseal portal vascular system passes through the sellar diaphragm, making the blood supply for the pituitary gland highly vulnerable to mechanical compression (Figure 2).

In addition to the intracranial swelling and increased pressure that occur during the evolving TBI-related process, other mechanisms of injury occur. Diffuse axonal injury (DAI) describes a process that occurs when two tissues of differing density experience external forces. These forces include rotational, acceleration and deceleration that occur with almost every injury such as falls, motor vehicle accidents, or assaults. When the brain suffers an external force, structures of differing densities and distances from the axis of rotation slide over one another. This phenomenon occurs between the gray and white matter within the CNS, resulting in DAI (stretching and tearing of tens of thousands of neurons). It is estimated that two thirds of DAI occur in areas where grey and white matter meet. Greater than half of all persons experiencing TBI suffer from DAI. The process of DAI explains much of the long term cognitive dysfunction following TBI [14]. Disturbances of attention, memory, and executive functioning are the most common neurocognitive consequences associated with this process [14,15].

Emergency medical personnel classify severity of injury according to the Glasgow Coma Scale (GSC). GCS grading is used to assess level of consciousness after injury based on eye response (scores of 1–4), verbal response (scores of 1–5) and motor response (scores of 1–6). Mild TBI, defined as a GCS between 12 and 15, typically represents a blunt trauma to head with short loss of consciousness. Moderate TBI is defined as a GCS of 9–11, and severe TBI as a GCS ≤ 8, with lower values indicating greater degree of injury (complete unresponsiveness to verbal, motor and no eye opening consistent with GCS of 3, as an example of most severe injury). Classification of injury based on GCS has limitations including inter-observer reliability, timing after injury, reduced meaning after repeated injury and assessment, as well as use of medications at the scene of the accident potentially altering scoring [16,17,18]. It is not yet clear how assessment by the GCS correlates with risk for hypothalamic-pituitary dysfunction. In addition, little data exist in the pediatric literature regarding the predictive value of the GCS, especially in very young, pre-verbal children [19].

## 3. Incidence of Endocrine Changes Following TBI: Adult Literature

Case reports showing association between TBI and hypopituitarism were originally published in the early 1900s. A pivotal clinical article by Benvenga [20] further confirmed the relationship of TBI-induced hypopituitarism. Overall, hypothalamic-pituitary dysfunction has been reported in 23% to 69% of adult patients, assessed at 12 months or more after moderate to severe TBI [21,22]. The most common endocrinopathies in adults after TBI are growth hormone deficiency (GHD) and hypogonadism. However, central hypothyroidism (TSHD), adrenocorticotropin hormone (ACTH) deficiency, diabetes insipidus (DI), as well as hyperprolactinemia have been reported. These deficiencies can occur either acutely or develop slowly over time (Figure 1). Isolated neuroendocrine abnormalities can be transient or permanent, with some studies suggesting that, when observed, complete pan-hypopituitarism tends to be permanent [23]. Most studies, but not all, report that the incidence of pituitary deficiency is not correlated with severity of injury as assessed by GCS. In cross-sectional evaluation of 126 adult TBI survivors (mean age 42.4y, 60% severe TBI, and 40% moderate TBI), 57% had endocrine deficiencies including GHD in 39.7% [24]. Prevalence of different pituitary hormone deficiencies depends on the mechanism of injury, degree of injury, time between injury and assessment, choice of endocrine testing modality, and definitions of deficiency [25].

In 23 patients aged 16–25 years, the incidence of pituitary abnormalities was 35% at three months and 30% at 12 months after TBI [23]. Some abnormalities resolved by 12 months, with other patients developing new pituitary hormone deficiencies at 12 months. The most common pituitary abnormality was GHD, followed by hypogonadism. In studies of endocrine deficiencies following TBI in adults, patient selection and dissimilar methodologies accounted for variations in overall prevalence and influenced the incidence of abnormality detected [15,22]. However, given the high incidence of TBI, even relatively low occurrence of endocrine deficiency would translate into a large number of individuals developing hypopituitarism after TBI. In GH-treated TBI survivors enrolled in a placebo-controlled study of GH therapy, cognitive function improved including processing speed, short-term memory, and executive function [26,27]. Thus, GHD may explain common complaints reported by recovering individuals including fatigue and cognitive challenges [15].

## 4. Acute Life Threatening Effects on Endocrine System in Children

Significant injury to the hypothalamic–pituitary axis from TBI may complicate medical management in the period immediately after injury. Acute changes in ability to release vasopressin, thyrotropin (TSH), or cortisol are examples. Diabetes insipidus (DI) is uncommon after TBI, but when present nearly always occurs early in the acute phase after injury. DI can be evident within hours to days after the injury, but, alternatively, the onset of DI may be delayed as long as 30 days after injury [28]. In a series of 19 children with brain-damage (12 after TBI), all with DI, only three patients survived, leading to the conclusion that DI may be an indicator of poor prognosis [29]. Regardless, permanent DI is rarely reported after TBI in children.

In the first few days after injury in a person with intact hypothalamic-pituitary function, cortisol levels would be expected to rise in response to the stress of injury and hospitalization. Admission cortisol levels within a few hours of injury in a person with an intact hypothalamic-pituitary axis tend to directly correlate with injury severity scores and neurologic outcomes. Lower cortisol levels are seen in milder injury and the highest levels in the most severely injured [30,31]. Thus, an inappropriately low cortisol level obtained acutely after severe injury may suggest secondary adrenal insufficiency (ACTH deficiency). In a prospective study of 80 adults evaluated acutely after TBI, there was a 53% incidence of adrenal insufficiency based on low cortisol levels, and an inverse association with injury severity [32]. Children may differ in their response in the acute phase after TBI, but reports are limited. In 37 children studied on the first, third, and seventh days after severe TBI, cortisol was elevated on day one, but 46% had a low cortisol on day three, suggestive of ACTH “fatigue” due to persistent stress [33]. In contrast, a retrospective study found no children with low cortisol levels acutely after TBI [34]. Patients with moderate to severe head injury should have their adrenal status evaluated acutely after injury (serum cortisol within the first 12 to 24 h). In the intensive care setting, a random cortisol level >25 µg/dL during the stressed state suggests adrenal sufficiency [28]. If children do not meet these criteria, short-term therapy should be provided with stress doses of hydrocortisone (30–100 mg/m^2^ HC) or other steroid that has both glucocorticoid and mineralocorticoid effect. Steroid therapy should be tapered as soon as the clinical status of the patient improves. Approximately one month after discontinuing steroids, assessment of the adrenal axis should be obtained with low dose ACTH stimulation testing.

Disturbances in thyroid function are commonly observed acutely after brain injury, characteristic of a state of non-thyroidal illness including low triiodothyronine (T3) and low normal thyroxine (T4), usually with a low-normal to mildly elevated level of TSH [35]. Clinical effects of hypothyroidism in acute critical illness could include lethargy, difficulty awakening, bradycardia, cardiac failure, or hypothermia.

## 5. Late Effects of TBI on Endocrine System in Children

By comparison with reports in adults, in children there is limited literature and research regarding longer term endocrine dysfunction after TBI (Table 1). Theoretically, pediatric patients may have an improved prognosis for neurologic recovery compared to adults with the same GCS scores, due to improved neuroplasticity [25]. Case reports have documented pituitary dysfunction including precocious puberty in children [36]. However, only a few studies have systematically evaluated the prevalence of endocrine dysfunction in children after TBI. Cross-sectional studies documented 16%–61% prevalence of hypopituitarism at 1–5 years after injury [23,34,37,38,39,40,41,42,43]. A number of reviews and commentaries have discussed the need for prospective studies of hypothalamic-pituitary function after TBI in children [44,45,46]. To date, only four prospective studies in the pediatric age group have been published regarding endocrine abnormalities after TBI [34,47,48,49].

Einaudi [34] prospectively studied 30 children (average age of 9 years old) at 0, 6, and 12 months after injury. At baseline after injury, the incidence of endocrine dysfunction was 23%. At six months, the incidence was 4%, with one patient having ACTH deficiency. At twelve months, the incidence was 10% (*n* = 2), with one patient having a new diagnosis of GHD, and one continuing to have ACTH deficiency. Combined with the retrospective data from the same study, the incidence of some degree of hypopituitarism was 10.4% at six months after injury.

Kaulfers [47] prospectively evaluated 31 children and adolescents for endocrine function after TBI. The incidence of any endocrinopathy in children who had moderate to severe head injury was 15% at one month after injury, 75% at six months, and 29% at 12 months after TBI. Interestingly, many of the endocrine abnormalities found in the first few months after injury resolved by one year. Loss of menstrual regularity was common in the first six months after injury in 22% of adolescent females, but all resolved by one year. Three children had water imbalance/diabetes insipidus, but all were transient and resolved by six months. Eight children (33%) had elevated prolactin, but all had resolved by 12 months. One case of secondary adrenal insufficiency at six months resolved by 12 months. Of thyroid abnormalities in the 13 children, all but two resolved by 12 months. Of 13% with GHD at six months, all but one resolved by 12 months. Four of six prepubertal age children developed precocious puberty, while 14% of the pubertal adolescents showed rapid pubertal progression. No risk factors such as GCS or radiographic findings were identified that could indicate which children would have endocrine abnormalities.

Casano-Sancho [48] prospectively followed 37 children for one year (60% severe TBI, 23 greater than six years old). Those older than six years underwent endocrine evaluation at three and 12 months after TBI. Of them, 11 of 23 (48%) had subnormal GH peak at three months that persisted in eight of 23 (34%) after one year. Interestingly, growth velocity was normal in all patients except for one, but all demonstrated significant increase in body mass index. Similarly, 10 of 23 had suboptimal cortisol response (7–16 mcg/dL) to glucagon initially, which normalized over time in seven of 10. Transiently abnormal thyroid function was noted in three patients, while no sustained pubertal abnormalities were observed.

**Table 1:** Pediatric literature review: Studies of endocrine function after pediatric traumatic brain injury.

Author; year [reference]	Study Method # of patients	Age at injury (yo), Time after TBI until time of study (m or y)	TBI Severity (GSC)	Testing methods	Overall Prevalence of dysfunction	Pituitary Dysfunction by hormone
Einaudi 2006 [34]	Prospective 30	Injury: 9.1 years old (0.25–15.5 yo)	6 severe 9 moderate 15 mild	Baseline: T0, T6 & T12 GHRH + Arginine Glucagon	T0: 7 of 30 T6: 2 of 26 T12: 2 of 20	T0: abnormal TFTs T6: low cortisol (2) T12: GHI (1), GHD (1)
Niederland 2007 [37]	Cross-sectional 26	Injury: 8.9 yo Time to study: 30.6 ± 8.3m	Mixed	Screening TBI *vs*. controls 1st GHST: L-DOPA 2nd GHST: ITT	60% dysfunction 42% diminished GH	l-DOPA:GH 16.8 *vs*. 32 (*p* = 0.003) ITT:GH 20.5 *vs*. 27 (*p* = 0.06) Cortisol: 19 *vs*. 26 (*p* = 0.002)
Poomthavorn 2008 [38]	Cross-sectional (Questionaire) 54	Injury: 9.7 yo (0.3–16.8) Time to study: 4.5 y (0.9–8.5)	All severe	Baseline: 29 of 54 Glucagon Stim if poor GV & low IGF (8 of 29)	16.6%	1 female precocious puberty 1 TSHD 2 gonadotropin def 3 partial ACTH def 2 GHD
Norwood 2010 [39]	Cross-sectional 32 pts	Injury: 12.7 yo Age at study (15.7 yo)	Mean: 5 Range: 3–15	Overnight GH (<5 ng/mL) AND Arginine/glucagon (<7 ng/mL)	34% failed either testing modality	5 of 32 failed both 6 of 32 failed overnight 10 of 32 failed GHST
Kaulfers 2010 [47]	Prospective 31	Injury: 11.6 yo	24 severe	Screening: baseline, 3, 6 & 12 m 6m: overnight GH & TSH, ACTH stim 12m: GHST (Arg + Clon or GHRH)	Baseline: 2 of 3 DI resolved 21% abn TFTs 3m: all DI resolved 24% abn TFTs 5 of 9 oligomenorrhea	6m: 13% low GH surge 46% low TSH surge 1 poor ACTH 12 m: 2 TSHD 1 GHD 3 males PP or rapid
Heather 2012 [40]	Cross-sectional 198	Injury: 1.7 ±1.5 yo Time to Study: 6.5 ± 3.2 y	27% severe 18% moderate 55% mild	Screening fasting Clonidine & Arginine (<5 mcg/L)	33% GH peak < 10 mcg/L 8% GH peak < 5mcg/L 9% poor ACTH response 1% PP	No treatment initiated. All demonstrated normal growth 5 of 18 repeat GHST- only 1 failed 13 of 17 with AI passed retesting
Auble 2013 [41]	Cross-sectional 14	Injury 0.5 yo (1–1.1) Time to Study: 2.5 y (2–9 y)	All severe: 11 required intubation 11 with seiz	Overnight TSH, GH sampling Fasting baseline and low-dose ACTH	86% abnormal labs or height <10%ile	Most common: elev. Prolactin Blunted TSH surge (<50% rise) 2 with poor GH surge
Bellone 2013 [42]	Cross-sectional 70	Injury: 8.1 ± 4.2 yo Time to Study: 1–9.1 y	19 severe 11 moderate 40 mild	Baseline & 12m if poor GV GHST (GHRH + Arg) at 12m	Screening: 4 cases 6m: 20 of 70 poor GV 12m: 13 of 20 poor GV	Baseline: TSHD & ACTH def (1) FSH/LH def (1) ACTH def (1) PP (1) 12m: 4 of 13 GHD Total: 10%
Casano-Saucho 2013 [48]	Prospective 37 pts	14 pts: age 0.2–2.3 yo 23 pts: age 7–19.9 yo	22 severe 7 moderate 8 mild	<6 yo: baseline at 12 m >6 yo: baseline & 2 dynamic tests 3m & 12 m (glucagon/clonidine <10 ng/mL)	3m: 11 of 23 GHD 10 of 23 ACTH 12m: 8 of 23 GHD 3 of 23 ACTH	<6 years old- no baseline or clinical abnormalities No sustained pubertal abnormality Transient thyroid 3 of 23
Salomon-Estebanez 2014 [43]	Cross-sectional 36	Injury: 3.3 yo Time to Study: 7.2 y	36.6% severe & moderate 63.4% mild	Screening; provocative testing if abnormal	4 low IGF markers 2 low cortisol	No dysfunction observed after clinical follow-up No provocative testing
Personnier 2014 [49]	Prospective 87	Injury: 6.7 yo (0.8–15.2)	All severe	Baseline + 1st GHST (betaxolol, glucagon or glucagon only) 2nd GHST at 9 m after TBI if 1st <7 ng/mL (arginine, insulin)	17% severe GHD 6 pts transient TFTs 1 with AI	1st GHST: 35 of 87 failed 2nd GHST: 27 of 33 failed (22 with normal IGF values) Only 6 demonstrated poor growth

Personnier [49] prospectively followed 87 children and adolescents hospitalized for severe TBI with follow-up endocrine assessment performed between 6–18 months after injury. Initial assessment demonstrated GH peak <7 ng/mL in 40%, six with low FT4, and one with low cortisol. Of the 33 patients that underwent repeat GH stimulation testing, 27 continued to demonstrate poor GH response with the majority having normal IGF-I markers (*n* = 22). On retesting, TSH deficiency was confirmed in two, and ACTH deficiency in one patient.

## 6. Changes in Endocrine Function According to Specific Deficiency

In long-term follow-up of children after TBI, issues of hypothalamic and/or anterior pituitary deficiency can have significant effects on quality of life especially with regard to growth and puberty. Case reports of children months to years after head injury reported poor growth, explicit GHD, precocious puberty, or failure to enter or progress through puberty. Likewise, adults experience chronic fatigue, loss of libido or amenorrhea, or overt gonadotropin deficiency in addition to a variety of neurocognitive complaints [12,50]. Onset of these symptoms may be insidious and confused with the post-concussive syndrome, so pituitary dysfunction may go unrecognized and untreated in adults and children after TBI [32].

It is crucial that medical providers recognize that a history of CNS injury of any etiology may lead to pituitary dysfunction. For instance, inflicted TBI/shaken baby syndrome is an under-recognized form of TBI. Pituitary screening was performed in 14 children who had suffered from severe inflicted injury in infancy [41]. Median age of injury was five months, while age at time of evaluation ranged from two to 9 years old (median 3.1y old). Of this group, 29% had height shorter than the 10%ile, and 57% demonstrated some endocrine dysfunction (excluding hyperprolactinemia) [41].

Inconsistent methods of defining hypopituitarism contribute to the technical challenges in diagnosing children after TBI. Degree of injury as scored by GCS, timing between injury and endocrine assessment, age at time of injury, and lack of consensus guidelines all contribute to wide variability of endocrine diagnosis and prevalence of endocrine deficiency in the literature. For instance, some of the studies used screening or basal hormonal concentrations, while others also used dynamic testing methods (stimulation tests). Different laboratory assays, stimulatory agents, and threshold values for diagnosis also contribute to differences in outcomes. For example, GH can be measured by several different types of assays with results that may differ three-fold (immunoradiometric assays (IRMA) *versus* radioimmunoassays (RIA) *versus* enzyme-linked immunoassays (ELISA)) [51]. In addition, many drugs used in the acute or chronic stages after injury (*i.e.*, anti-seizure medication, antidepressants, and antipsychotics) may influence results by interfering with neuroendocrine function.

### 6.1. GH Deficiency

Subtle GH deficiency can be difficult to identify, especially in adults (who are no longer growing). Screening tests such as IGF–I and IGFBP3 can be helpful in excluding deficiency if the patient‘s results are above average for age (high sensitivity), but poor specificity as the adult data revealed that only 17%–30% of adult GHD subjects exhibited a low IGF-I level [39]. Children with GHD can have IGF–I results below normal limits or in the lower half of the normal range [52]. GH stimulation tests identify children and adults who have clear–cut GHD but may miss persons with partial GHD [53]. Overnight (12 h) spontaneous GH secretion has been found to be lower than normal in some children after cranial irradiation (another example of hypothalamic injury), and can indicate a subtle GH defect [54]. A 6 h sampling of GH levels, from 2200 to 0400 h (normative results [55]), can accurately demonstrate a child’s ability to release GH, and is less invasive than a 12 h sampling period, although measurement of overnight secretory pattern of GH for clinical diagnosis remains controversial.

In adults and children who have suffered TBI, GHD is one of the most common endocrine deficiencies [21,22,56,57,58,59]. Among adult patients evaluated one year or more after TBI, the reported incidence of GHD varies between 2% and 57%. In a cross-sectional study of 126 adults evaluated on average 5.8 years after TBI, 57% were found to have GHD or GHI (GH insufficiency, defined as response to insulin-induced hypoglycemia between 3–10 ng/mL) associated with IGF-I SDS < −2.0 [24]. Discrepancies among studies can in part be explained by differences in the type of testing used, different stimulating agents, and different diagnostic criteria. Klose [60] assessed the prevalence of GHD in adults while assessing methodologic bias. Healthy adult control subjects were tested for GH response to stimulation tests to establish “local or regional” diagnostic criteria (defined by less than 2.5th percentile and compared to Danish national standards. Comparing national and local criteria, the prevalence of GHD varied more using combined testing: insulin tolerance test (ITT); pyridostigmine-GHRH or GHRH-Arginine agents. The prevalence defined by local cutoffs was 11.8%; when applying the national Danish criteria, incidence increased to 18.9%. In contrast, the incidence of GHD using Insulin tolerance testing (local criteria ≤2.6, national criteria of ≤3 mcg/L) was similar at 5%.

Among the prospective studies in children, Einaudi [34] reported one of 30 patients with GHD at 12 months (3%). Norwood [39] studied 32 children recruited from the pediatric TBI clinic using both a low overnight GH sampling surge and response to arginine/glucagon testing (Table 1). Five of 32 subjects (16%) failed to have overnight GH peak above 5 ng/mL and 10 of 32 (22%) had a stimulated peak GH response less than 7 ng/mL. Collectively, 34% of subjects exhibited insufficient GH secretion by one of the two tests. Although IGF-I levels tended to be lower in those with GHD, they were not significantly lower than in those subjects without GHD [39]. Of 191 children studied 6.5 years after injury (mean age at injury 1.7 ± 1.5 years old), 65 (34%) demonstrated a subnormal GH response of less than 10 mcg/L, while 16 had GH peak less than 5 mcg/L to dual stimulation with clonidine and arginine [40]. Despite relatively high incidence of insufficiency or deficiency, both IGF-I and IGFBP3 were within the normal range and all subjects demonstrated normal interval growth velocity [40]. Bellone [42] assessed whether measurement of height velocity is a useful screening marker in children following TBI, finding that 20 of 70 patients had a six-month growth velocity <25th percentile (none below 3rd percentile). Reassessment at 12 months revealed that 13 of the 20 patients with documented poor velocity continued to demonstrate velocity below the 25th percentile, with four having impaired GH peak after GHRH plus Arginine stimulation [42]. The prospective study by Casano-Sancho [48] noted that 11 of 23 patients ≥6 y old had a subnormal GH peak response to glucagon and clonidine three months after TBI that persisted in eight of 23 (34%) after 1 year. Growth velocity was normal and IGF-I levels were normal in all patients except one; however, BMI increased significantly in those with confirmed GHD [48]. Salomon-Estebanez [43] also suggested that IGF-I values and growth velocity were poor screening tools for detection of TBI-induced GHD. They recruited 36 children (age at injury 3.8 y, age at study 7.2 y) to undergo systemic examination with baseline screening pituitary testing. Four subjects with IGF-I level below the 2.5%ile despite normal IGFBP3 levels were followed for one year. On retest all four subjects demonstrated spontaneous increase in IGF-I levels and normal growth velocity, therefore no subjects underwent stimulation testing [43].

Prospective study of GH stimulation testing in 87 children at three months after TBI demonstrated that 40% (35 subjects) had subnormal GH response <7 ng/mL to betaxolol and glucagon [49]. Growth follow up 4.9 months later in those who demonstrated poor stimulated GH response revealed normal mean growth velocity in most, while 31% had poor growth response. Only 18% of confirmed GHD subjects had IGF-I < −2 SDS. Thus, IGF-I levels were not sensitive as screening tools for GHD [49].

Regardless of testing method used, these studies show that GHD remains an important problem that requires clinical follow-up. In addition, these results suggest that GHD can be transient in children after TBI. Children should be followed for longer than 12 months after TBI, as one year may be too early to demonstrate a decline in height velocity.

### 6.2. Gonadotropin Deficiency

Gonadotropin-releasing hormone (GnRH) deficiency is the second most common endocrinopathy after TBI. Prospective studies in adults show a much higher prevalence of GnRH deficiency acutely after injury than in the long-term, suggesting that hypogonadism may be a temporary adaptive response to injury [61]. Kokshoorn [62] studied 112 adults (mean age of 48 y, who had been hospitalized for average of 11 days, 33% with severe injury based on GCS) at a mean duration of follow up of four years). Morning luteinizing hormone (LH) and follicle stimulating hormone (FSH) screening confirmed GnRH deficiency in one individual. However, neither testicular characteristics nor menstrual irregularity were factored into the screening protocol, thus the deficiency was likely under-represented [63]. Gonadal axis dysfunction ranged from 4%–37% with follow up after injury varying from less than one year to 12 years [15].

In the pediatric population, assessment of changes in pubertal progression is often challenging (rate of change in physical signs of puberty or consistency of breast or testicular tissue). In addition, normative ranges for laboratory values change as puberty progresses. Among retrospective pediatric studies, Poomthavorn [38] found that 4% of children had hypogonadism within the first year of injury, either transient or permanent, with no new cases of hypogonadism found at more than eight months after injury. Einaudi [34] evaluated one cohort cross-sectionally and another prospectively. Retrospectively, the study found a 9% incidence of hypogonadism at one to four years after injury in children, while prospectively, the study found no cases of hypogonadism at one year after injury [34]. Observations by Kaulfers [47] also suggested that hypogonadism is usually transient. One patient clinically presented with hypogonadism within one month of injury, and by three months he had recovered. Female patients has secondary amenorrhea which resolved by one year after injury. One new case of GnRH deficiency was evident at one year, highlighting the need for these children to be closely monitored for at least one year after injury [47]. Of note, it is not possible to diagnose a prepubertal age child with hypogonadism, therefore follow-up into the pubertal age range is necessary.

### 6.3. Precocious Puberty

Precocious puberty (PP) after TBI, as opposed to hypogonadism, is a phenomenon unique to children. PP may go unrecognized and untreated in children after TBI. The literature has several case reports of PP in children after TBI. Among retrospective pediatric studies, Poomthavorn [38] identified one child (2%) with PP four years after TBI, and Einaudi [34] found one child (5%) with PP five years after injury. In the cohort evaluated by Auble, two of 14 had PP onset five to six years after nonaccidental TBI (Rose, unpublished). Prospectively, Kaulfers [47] found a high incidence of rapid and/or precocious puberty in children after TBI. Overall, four children (16%) had a rapid tempo of puberty by six months after injury, and all of them continued to progress in puberty at the 12 month visit. Two of them required GnRH agonist therapy to slow down the progression of puberty [47]. Heather identified PP in one of 116 boys and in one in 82 girls [40]. Personnier [49] prospectively followed 87 children and adolescents, performing endocrine assessment between six and 18 months after injury including clinical pubertal staging and baseline serum gonadotropin concentrations if older than 10y in girls or 11y in boys. No patients experienced precocious or delayed puberty [49]. Only one of 16 peripubertal children followed for one year after TBI failed to respond to GnRH testing [48].

Brain injury may interfere with the typical prepubertal inhibition of gonadotropin release. Gonadotropin release is actively suppressed in the prepubertal years. The mechanism of early or rapid puberty after TBI may involve loss of neural inhibitory influence of gamma-aminobutyric acid (GABA) on the GnRH pulse generator, or alternatively loss of inhibitory effects on the *N*-methyl-d-aspartate (NMDA) receptors resulting from the hypothalamic-pituitary injury [63,64]. This process is similar to that involved in development of precocious puberty after meningitis, hydrocephalus, brain tumors, or encephalopathy.

### 6.4. ACTH Deficiency

Assessment for acute adrenal insufficiency following TBI is challenging. Distinguishing pathophysiological changes due to brain injury *versus* the body’s response to intensive care treatment and medication is compounded by the uncertainty of “appropriate post-injury” cortisol levels. The acute adrenal response to TBI shows a significant cortisol elevation after moderate TBI, while after severe TBI there may be inappropriately low cortisol level on day 7 [65]. Individuals with lower cortisol required longer duration of assisted ventilation and had worse physiology scores SAPS II and APACHE (estimates of hospital mortality). Interestingly, 23 of the 68 subjects underwent low dose ACTH testing 2 y later with 43% (*N* = 10) demonstrating insufficient response (<18 mcg/dL) [65]. Possible explanations include ACTH deficiency or posttraumatic stress disorder-associated insufficiency. Selection bias may artificially lower the reported incidence of ACTH deficiency in the acute setting due to mortality. Cortrosyn secreting cells are centrally located in anterior pituitary, thus may be relatively protected from the primary and secondary injuries after TBI. Limitations that apply to assessment of other hypothalamic-pituitary axes (different testing agents, doses and diagnostic criteria) also factor into determination of the frequency of ACTH deficiency following TBI. Assessment methods in different publications include basal cortisol concentrations, low-dose ACTH stimulation (1 mcg), standard dose ACTH stimulation (250 mcg), metyrapone, glucagon, and insulin hypoglycemia. Results were less consistent among studies when basal cortisol levels were used, than when the low-dose ACTH test was used to make the diagnosis of secondary adrenal insufficiency after TBI [29]. A low-dose ACTH stimulation test (1 mcg/m^2^, with peak cortisol obtained at 20–30 min) can be used to evaluate for hypothalamic or pituitary adrenal insufficiency [66]. Meta-analysis of ACTH testing evaluating primary data from 13 studies indicated that the mean basal morning cortisol in patients without adrenal insufficiency was 13 µg/dL. After low-dose stimulation with cortrosyn, cortisol levels over 22 µg/dL confirmed adrenal sufficiency [66].

Kaulfers [47] found no cases of low basal cortisol levels before six months after injury, using relatively strict criteria for diagnosis (cortisol <5 mcg/dL). Basal cortisol concentrations did not correlate well with the peak cortisol response to low-dose ACTH stimulation test at the six month visit. At that time, only one patient had a low basal cortisol (3.3 mcg/dl) but had a normal stimulated cortisol level (26.3 mcg/dL), while another patient had a normal basal cortisol (11.9 mcg/dL) but a borderline low stimulated cortisol (18.1 mcg/dL).

Poomthavorn [38] demonstrated similar results in retrospective study of 54 patients. Only one of 15 patients tested had a low basal cortisol level, but that patient had a normal stimulated cortisol level on a low-dose ACTH stimulation test [38]. In 26 children tested after TBI, stimulated cortisol levels were lower than in healthy controls, using insulin tolerance testing [36]. In a prospective study by Einaudi, two patients had low basal cortisol levels at six months after injury, only one of whom failed a glucagon stimulation test; ACTH deficiency was confirmed in that patient at 12 months after TBI [34]. Heather evaluated cortisol response to low dose ACTH testing, with suboptimal cortisol response (<500 nmol/L) found in 17 of 198 (9%) participants. Subsequently, 13 of the 17 demonstrated normal response on repeat testing [40].

Bellone [42] evaluated 70 children at 1–9.1 years after TBI (mean age 8.1 ± 4.2 y). ACTH deficiency was found in four children: one with ACTH and TSH deficiencies, one with ACTH and FSH/LH deficiencies, one with ACTH deficiency and PP, and one with isolated ACTH deficiency. Overall at one year after TBI, seven patients had one deficiency or multiple hypothalamic-pituitary dysfunction (10%) [42]. Likewise, 10 of 23 children over six years old demonstrated suboptimal cortisol response to glucagon at three months after TBI, with all but three normalizing by 12 months after TBI [48]. Endocrine screening of 36 patients (mean age 7.2 years, mean duration since TBI 3.3 years) identified six who required further investigation: four with low IGF-1 levels and two with low cortisol. Subsequent insulin tolerance testing revealed normalization of ACTH-cortisol axis [43]. Of 87 children followed prospectively, one individual experienced symptoms of cortisol deficiency confirmed by low dose ACTH testing [49]. Of note, each pediatric study used different standards for diagnosis of ACTH deficiency, thus it is difficult to draw conclusions about true incidence. However, each study has found only a few patients with persistent adrenal insufficiency. Therefore, even though development of ACTH deficiency is a possibility and is concerning as a life-threatening hormone deficiency, it is not as common as GHD or GnRH deficiency in children after TBI.

### 6.5. Central Hypothyroidism

Disturbances in thyroid function are commonly observed acutely after brain injury, characteristic of a state of non-thyroidal illness including low T3, elevated reverse T3, and low normal T4, usually with a low-normal (but sometimes elevated) level of thyrotropin (TSH) [35]. Thyroid levels in central hypothyroidism can be similar to those in non-thyroidal illness. Thus, diagnosing central hypothyroidism following traumatic brain injury can be challenging. Kaulfers found that 21% had a low Free T4 (FT4) at baseline and 24% at three months after TBI [47]. Einaudi found similar results with 23% of children transiently having low Free T3 acutely after injury [34]. Among 198 children at a mean of 6.5 + 3.2 years after injury, only one had laboratory results suggestive of TSH deficiency. However, this child’s thyroid function normalized later without therapy [40]. In 70 children evaluated 1 to 9.1 years after TBI, one had TSH deficiency associated with panhypopituitarism [42]. Among 87 children studied 9.5 + 3.4 months after injury, six had central hypothyroidism [49]. Interestingly, Kaulfers found that 11% had an elevated TSH at baseline after injury [39]. Similarly, three of 37 children followed prospectively after TBI had elevated basal TSH with FT4 within the normal range; all these values normalized at 12 months after injury [48]. The suggested mechanism for central hypothyroidism with elevated TSH is abnormal glycosylation of the TSH alpha and beta subunits, thus the TSH secreted is not biologically active [67,68].

The current most sensitive method for confirming central hypothyroidism (TSHD) requires recognition of the circadian pattern of TSH secretion [68]. A significant rise in TSH occurs overnight in normal children and adults, termed the TSH surge [69]. Alternatively, clinicians can screen for the TSH surge using a TSH at 0800h compared to a TSH after 1000h or in the afternoon (AM to PM TSH ratio). AM to PM TSH ratios under 1.3 associated with a low normal FT4 are consistent with blunting of the TSH surge, confirming central hypothyroidism [68].

Failure to recognize and treat TSHD can result in less than optimal state of health and poor growth. In a prior report of children with short stature, an incidence of approximately 13 per 100 short children had a blunted TSH surge associated with low or low–normal Free T4 in the absence of any other pituitary hormone disturbance. The incidence was 33% of those with both with height shorter than –2 SD and a Free T4 in the lowest third of the normal range. These children showed a significant increase in growth velocity during levothyroxine therapy compared to the growth response of short children with FT4 in the lowest third of normal who had a normal TSH surge [70]. An abnormal TSH surge was identified in 46% of patients at six months after TBI, and most (72%) of them had a Free T4 in the lowest third of the normal range. The TSH surge continued to be abnormal in 10% of the patients at 12 months [47]. Thus, central hypothyroidism can be a transient or persistent deficiency. Other pediatric TBI studies found an incidence of TSH deficiency at one year after injury (5%–12%) similar to that observed in adult TBI studies (2%–22%) [21,28,29,30]. In pediatric survivors of inflicted TBI (shaken baby syndrome, SBS), testing of 14 children at median of 3.1 years after TBI found six of them to have blunted TSH surge [41].

Another model of CNS injury to the hypothalamus is cranial irradiation. TSH surge was found to be abnormal in 43% of referred poorly growing childhood cancer survivors [71]. Diagnosis of central hypothyroidism in many patients after injury would have been missed by only checking screening TSH and FT4.

### 6.6. Hyperprolactinemia

Elevated prolactin (PRL) reflects probable pituitary stalk injury with disruption of the inhibition of prolactin secretion by dopaminergic signals from the hypothalamus. However, PRL secretion is an acute phase reactant; mild elevation occurs during illness or stress such as during phlebotomy making the clinical diagnosis challenging. However, PRL elevation from stalk injury is often higher than induced by emotional stress. Hyperprolactinemia may occur after hypothalamic or pituitary injury in both adults and children, but is usually transient and resolves within the first year after injury. Two retrospective studies in children each reported one child with elevated PRL one to two years after injury [37,38]. Both children had PRL elevation associated with other pituitary deficiencies including transient hypogonadism. There were no reports in the pediatric literature of elevated PRL more than two years after injury. In prospective study, PRL elevation was one of the most common abnormalities observed at both three and six months after injury, even after eliminating those patients on medications that cause hyperprolactinemia [47]. However, all cases resolved by 12 months after injury. Among 14 children after inflicted injury (mean age of injury five months old, age at study of 3.1 years), the most common abnormality noted was mildly elevated prolactin (>14.7 ng/mL) in 64% [41]. Half of the 14 had two or more abnormalities (abnormal TSH surge in 43%, short stature in 29%, and low GH peak in 17%). The threshold for symptoms from TBI-induced hyperprolactinemia has not been determined.

### 6.7. Diabetes Insipidus (DI)

In prospective studies of children following TBI, only one of 87 exhibited signs of posterior pituitary dysfunction (polyuria, polydipsia) at six months but had appropriate response to fluid deprivation [49]. Of 31 children prospectively evaluated after severe TBI, 10% had transient DI but all cases resolved [47]. In studies with a wider spectrum of injury based on GCS, most describe transient water imbalance recognizing the possibility of selection bias (that the most severe and highest risk subjects do not participate in studies). Likewise, cross-sectional studies may exhibit a different form of selection bias as perhaps those that suffered DI had resolved prior to enrollment. Overall and fortunately, persistent DI appears to be a potential but rare phenomenon in children after TBI.

### 6.8. Predictors of Endocrinopathies

It has been estimated that 43% of adult patients discharged from hospitals after TBI have long-term disabilities [15]. Key points were that, after TBI, individuals with endocrine dysfunction have poorer outcomes; selection bias and dissimilar methodologies may account for varied estimates of prevalence of dysfunction; and regardless of prevalence, the high incidence of TBI equates to a large number of individuals in the community with hypopituitarism. Numerous factors limit determination of precise prevalence of endocrinopathy after TBI: selection bias, mechanism and degree of injury, timing between injury and evaluation, diagnostic tools used, diagnostic criteria applied. Prevalence of endocrinopathies at 12 months has not been shown to correlate with severity of injury as measured by the Glasgow Coma Score [24,47,49]. Radiographic findings on CNS imaging did not reliably predict presence or absence of endocrinopathies at 12 months after TBI [24,47,49]. The need for intracranial drain/surgical intervention had the greatest odds ratio as a potential risk factor for pituitary dysfunction [24]. Patients with persistent endocrinopathies at one year reported a higher incidence of weight gain, mood disturbances and altered appetite than those without hormone deficiencies [47,59]. Thus, if children report fatigue, cold intolerance, poor growth, altered puberty, mood disturbances, or altered appetite control after TBI, they should be considered for an endocrine evaluation.

## 7. Time Course of Changes in Endocrine Function over Time after TBI

In survivors, endocrine deficiencies after TBI may present acutely or develop slowly over months to years. In 126 adults after TBI, 32% of those followed beyond one year of injury demonstrated a significant change in pituitary function: 19.5% developed deficiencies over 44 months, while 12.2% demonstrated resolution during an average follow up of 52 months [24]. Mechanisms described above explain how both acute and slowly progressive hypothalamic-pituitary dysfunction can occur. Most of the articles described above evaluated children after moderate to severe TBI, thus presumably mild injuries have a lower incidence of endocrine deficiencies. Of course there is little data in the literature about endocrine function after mild injury, so the incidence is not known.

Pituitary hormone deficiencies can have a profound impact on a child’s ongoing development and long-term recovery. In case reports of pituitary deficiencies after head injury in children, most were not diagnosed until years after the initial injury, leading to unrecognized poor growth and/or puberty [46]. Acquired GHD in childhood can impair adult height as well as body composition if there is a delay in diagnosis and treatment [72]. Consistent with adult literature, the most common endocrinopathies after TBI in children are GHD and hypogonadism. Hypogonadism may not be apparent in prepubertal age children and recognition requires a high index of suspicion in early adolescence. In addition, children may develop precocious puberty which can obscure recognition of compromised growth velocity caused by other pituitary deficiencies (TSH, GH). Normal pre-pubertal growth velocity is five to seven cm/year and pubertal growth velocity can be ≥10 cm/yr.

There can be considerable difficulty differentiating behavior changes and endocrine deficiency after TBI, from typical adolescent behaviors. Vague nonspecific signs and symptoms like chronic fatigue, gradual unexplained weight gain and decreased stamina could be suggestive of evolving deficiencies. Even with no evidence of endocrinopathies, there can be behavior change, changes in scholastic abilities and impaired executive function; likely secondary to the numerous shearing forces that severed tens of thousands of neurons between the gray and white matter within the CNS. Cognitive rehabilitation may help to improve memory impairment, attention deficits, communication skills and executive function [73].

## 8. Conclusions/Recommendations

In a consensus conference in 2005, systematic endocrine screening was recommended for all patients after moderate to severe TBI, because those found to have hormone deficiencies benefit from appropriate hormonal replacement [11]. Endocrine dysfunction in children after TBI is common, can evolve after injury, resolve, or persist. Acerini *et al*. provided review of the literature in 2006 [74] with conclusions and recommendations similar to our original review in 2012 [75]. Recognizing that most, but not all, endocrine abnormalities resolve by one year after TBI, up to 38% of endocrine deficiencies persist following severe injury. The review by Tanriverdi *et al*. [22] focused on pituitary dysfunction after TBI in adults, including effects on cognitive impairment, metabolic consequences of hypopituitarism, screening and diagnostic methods, and which patients should be screened [22].

We agree with their recommendations regarding screening patients who had complicated mild TBI for ACTH deficiency immediately after injury and at hospital discharge (if patient required greater than 24 h hospitalization) [22]. At six months, patients should be reassessed for ACTH, TSH and FSH/LH deficiencies with appropriate hormone replacement when indicated. At one year after injury, patients should be assessed for GH deficiency and treated if found to have deficiency. If no hormone deficiencies are found at 12 months, patients should be reassessed annually through the fifth year for hypopituitarism.

Focusing on the pediatric population, it is important to monitor growth velocity and pubertal progression, recognizing that individuals with GH deficiency during puberty will demonstrate pre-pubertal growth velocity. A pediatric endocrinologist will be able to recognize subtle clinical differences in growth velocity and pubertal examination that may detect deficiency. Endocrine deficiencies in childhood can lead to increased morbidity and lower quality of life (Table 2). It is critical for the endocrinologist to work closely with intensive care and rehabilitation colleagues to help ensure appropriate and timely referrals occur to ensure systematic attention to diagnosis, and initiation of therapy of endocrine abnormalities early after TBI.

**Table 2:** Recommended screening tests for traumatic brain injuries (TBI)-induced hypopituitarism to be done acutely, 3 months, 6 months, and yearly after injury (modified from reference 74).

Hormone test	Time of draw
Serum cortisol	800 h
Free thyroxine (FT4)	800 h
Thyrotropin (TSH)	800 h and 1600 h
Insulin-like growth factor (IGF-I)	800 h
Prolactin	800 h
Persons in puberty or of pubertal age: Follicle-stimulating hormone (FSH)	800 h
luteinizing hormone (LH), testosterone or estradiol	
Persons with polyuria: urine specific gravity, Na and plasma osmolality	After 12 h fasting
**Clinical Assessment**	
Height measurement and growth velocity (yearly)	
Pubertal Staging (yearly)	
Weight (yearly)	
Review of Systems (yearly): delayed puberty, lack of energy/stamina, reduced muscle mass, decreased bone density, changes in mood or scholastic decline	

Physicians must be mindful of previous CNS insults (inflicted injury or other trauma, serious CNS infections or medical treatments, or recurrent mild to moderate concussions) that can place an individual at risk for central endocrinopathies. If history confirms prior injury, providers must perform diligent screening including regular height measurements, screening laboratory testing, and surveillance for pubertal development. In the absence of endocrine and growth surveillance, years may pass before a correct diagnosis is made and endocrine treatment started, leaving a significant impact on quality of life [47]. Without a high index of suspicion, accurate historical information and serial examinations, some patients may never have their endocrine deficiencies identified. We recommend on-going endocrine surveillance at six and twelve months after TBI, to document resolution of temporary abnormalities and to ensure early intervention for persistent or late-occurring endocrinopathies.

## Abbreviations

ACTH: adrenocorticotropic hormone
BMI: body mass index
CNS: central nervous system
DAI: diffuse axonal injury
DI: diabetes insipidus
FSH: follicle-stimulating hormone
FT4: free thyroxine
GCS: Glasgow Coma Scale
GH: growth hormone
GHD: GH deficiency
GHRH: GH-releasing hormone
GHST: GH stimulation test
GnRH: gonadotropin-releasing hormone
GV: growth velocity
IGF-I: insulin-like growth factor-I
IGFBP3: IGF-binding protein 3
ITT: insulin tolerance test
LH: luteinizing hormone
PP: precocious puberty
PRL: prolactin
T3: triiodothyronine
T4: thyroxine
TBI: traumatic brain injury
TSH: thyroid stimulating hormone
TSHD: TSH deficiency or central hypothyroidism
